# Multiscale imaging of polarity in bovine oviductal organoids

**DOI:** 10.3389/fcell.2026.1859547

**Published:** 2026-06-09

**Authors:** Brandi Dunn, Mindy A. Meyers, Scott Burlingham, Tatsuya Morisaki, Anza Darehshouri, Ahmed Gad, Riley Thompson-Brandhagen, Timothy J. Stasevich, Fiona K. Hollinshead

**Affiliations:** 1 Animal Reproduction and Biotechnology Laboratory (ARBL), Department of Biomedical Sciences, College of Veterinary Medicine and Biomedical Sciences, Colorado State University, Fort Collins, CO, United States; 2 Animal Reproduction and Biotechnology Laboratory (ARBL), Department of Clinical Sciences, College of Veterinary Medicine and Biomedical Sciences, Colorado State University, Fort Collins, CO, United States; 3 Cellular Engineering and Mechanobiology Laboratory (CEML), Department of Mechanical Engineering, Translational Medicine Institute (TMI), Colorado State University, Fort Collins, CO, United States; 4 Department of Biochemistry and Molecular Biology, Colorado State University, Fort Collins, CO, United States; 5 Department of Cell and Developmental Biology, University of Colorado, Aurora, CO, United States

**Keywords:** confocal microscopy, electron microscopy, epithelial polarity, multiscale imaging, organoids, oviduct, reproductive biology, TIRF microscopy

## Abstract

Organoids are three-dimensional cell culture systems that recapitulate key structural features of their tissue of origin; however, interpretation of organoid architecture is inherently dependent on the imaging modality used. In organoids cultured within extracellular matrix (ECM), cells typically adopt an apical-in/basal-out configuration in which the apical surface faces an enclosed lumen, limiting direct visualization and access to the luminal surface. Experimental manipulation of polarity enables the generation of defined structural stages that can be leveraged to evaluate how imaging modalities resolve epithelial organization. In this study, ECM-embedded (apical-in) and suspension-cultured after ECM removal (apical-out) bovine oviductal organoids were used as controlled structural models for comparing imaging techniques across spatial scales. Apical-out organoids were generated by ECM removal and centrifugation, exposing the luminal epithelial surface. Using a multiscale imaging approach—including brightfield and time-lapse microscopy, confocal immunofluorescence with line-scan analysis, total internal reflection fluorescence microscopy (TIRF), and electron microscopy—we assessed how each modality captures distinct features of the epithelial surface. Brightfield imaging enabled rapid assessment of overall morphology but did not resolve membrane orientation, while time-lapse imaging revealed differences in organoid dynamics and surface behavior. Confocal microscopy demonstrated redistribution of cortical F-actin and overall epithelial organization within intact structures, whereas TIRF microscopy provided surface-restricted visualization consistent with external accessibility of actin-rich surface structures. Electron microscopy further resolved ultrastructural features, including microvillar projections, supporting differences in membrane-surface orientation at nanometer resolution. Across modalities, consistent structural differences between apical-in and apical-out organoids highlight how polarity-defined states influence the interpretation of organoid architecture. Collectively, this study presents a multiscale imaging framework for characterizing epithelial organization in organoid systems and demonstrates how polarity-defined structural states can be leveraged to improve interpretation across imaging modalities. These findings provide a foundation for standardized structural characterization of organoids and support their application in reproductive biology, including studies of epithelial–embryo interactions and luminal signaling.

## 1 Introduction

Epithelial cell polarity refers to the asymmetric organization of cellular structure and function along the apical–basal axis, a defining feature of epithelial tissues *in vivo*. The apical membrane, often characterized by microvilli and cilia, faces the lumen or external environment, while the basolateral surface interfaces with neighboring cells and the underlying extracellular matrix (ECM). This organization enables compartmentalization of cellular processes, formation of selective barriers, directed transport of ions and nutrients, and coordinated signaling between the luminal and stromal environments (Bryant and Mostov, 2008; Akhtar and Streuli, 2013; Rodriguez-Boulan and Macara, 2014).

Three-dimensional organoid culture systems have transformed the study of epithelial biology by enabling epithelial cells to self-organize into physiologically relevant tissue structures *in vitro* (Lancaster and Knoblich, 2014; Clevers, 2016; Rossi et al., 2018; Velasco et al., 2019; Kim et al., 2020; Sato et al., 2023). The apical–basal axis in organoids is established and maintained through tightly coordinated interactions among cell–cell junctions, cytoskeletal organization, and extracellular matrix (ECM) derived cues (Bryant and Mostov, 2008; Rodriguez-Boulan and Macara, 2014). Tight junctions act as a boundary separating apical and basolateral identity (Yu et al., 2005; Akhtar and Streuli, 2013; Rodriguez-Boulan and Macara, 2014).

When epithelial cells are embedded in laminin-rich ECM, such as Matrigel or similar basement membrane extracts, they typically form polarized, cystic structures in which the apical membrane faces a central lumen while the basolateral surface interfaces with the surrounding matrix. The configuration commonly referred to as “apical-in” closely resembles the *in vivo* organization in many glandular and tubular tissues, including the intestine, oviduct, and endometrium, as well as lumen-forming epithelial organoids derived from solid organs such as the liver and pancreas (Rodriguez-Boulan and Macara, 2014; Clevers, 2016; Co et al., 2019; Boj et al., 2015; Harrison et al., 2021). However, this configuration presents a key experimental limitation: the luminal surface is enclosed and not readily accessible for direct interrogation. As a result, investigation of luminal processes, including secretion, host-pathogen interactions, and epithelial signaling, is constrained in conventional organoid systems (Rossi et al., 2018; Co et al., 2019; Harrison et al., 2021; Jelinsky et al., 2023).

Multiple research groups have demonstrated that epithelial organization in organoid systems can be experimentally manipulated to generate “apical-out organoids”, in which the apical membrane faces the external culture environment (Co et al., 2019; 2021; Gjorevski et al., 2022; Kakni et al., 2022; Fiorotto et al., 2023; Yousef Yengej et al., 2023; Moss et al., 2025). Two main approaches are commonly used to generate apical-out organoids: removal of ECM support followed by suspension culture, or modulation of soluble signaling pathways. ECM removal and suspension culture have been associated with the reorganization of epithelial architecture and outward-facing apical features (Co et al., 2019; 2021). Similar structural transitions have been observed across diverse organoid systems, highlighting the plasticity of epithelial organization in response to changes in microenvironmental context (Parigoris et al., 2022; Fiorotto et al., 2023; Yousef Yengej et al., 2023). These changes are thought to reflect disruption of ECM-derived polarity cues, including integrin-mediated signaling involved in maintaining basal membrane organization (Myllymäki et al., 2011; Akhtar and Streuli, 2013; Rodriguez-Boulan and Macara, 2014; Koike et al., 2022).

A second approach involves modulation of soluble signaling pathways. Lipid mediators such as lysophosphatidic acid (LPA) and sphingosine-1-phosphate (S1P) can promote apical-out organization via GPCR–Rho signaling pathways that influence cytoskeletal dynamics and membrane positioning (Hu et al., 2021; Tidball et al., 2025). In this context, polarity-associated structural states are an active reorganization of epithelial architecture in response to extracellular signaling cues.

Reproductive epithelial tissues provide a particularly relevant context for investigating epithelial organization. The oviductal epithelium contains diverse secretory and multiciliated cells whose apical surfaces regulate key physiological processes, including gamete transport, fertilization, and early embryonic development (Coy et al., 2012; Maillo et al., 2016; Rizos et al., 2016; Menjivar et al., 2023; Thompson-Brandhagen et al., 2026). Organoid models derived from reproductive tissues—including endometrial and trophoblast organoids—have provided powerful platforms for studying implantation biology, host-pathogen interactions, and endocrine signaling (Boretto et al., 2017; Turco et al., 2017; Fitzgerald et al., 2019; Gu et al., 2020; Kim et al., 2020). These systems are typically maintained in an apical-in configuration, limiting direct access to the luminal epithelial surface. To date, these studies have primarily focused on establishing epithelial accessibility following polarity inversion (Ahmad et al., 2023; Zhang et al., 2025; Zhou et al., 2025; Thompson-Brandhagen et al., 2026) rather than systematically evaluating how epithelial organization is interpreted across imaging modalities. As a result, a comprehensive framework for characterizing polarity-associated structural reorganization in reproductive organoid models remains lacking.

Importantly, assessment of epithelial polarity in organoid systems is often inferred from morphological features or limited marker localization and is further complicated by technical constraints inherent to imaging three-dimensional structures. Limitations in optical penetration, resolution, and sample preparation can affect how epithelial architecture is visualized and interpreted across spatial scales. As a result, a single imaging modality may provide only a partial representation of epithelial organization.

The aim of this study was not to define the mechanisms of polarity reorganization, but to use polarity-defined organoid states as a controlled framework for evaluating how different imaging methods capture epithelial structure across spatial scales (Co et al., 2019; Kakni et al., 2022). By directly comparing ECM-embedded (apical-in) bovine oviductal organoids and organoids following ECM removal (apical-out) across complementary imaging modalities, we establish a multiscale reference for interpreting organoid architecture and highlight the strengths and limitations of each approach in resolving epithelial organization. This framework provides a basis for more consistent structural characterization of organoid systems and supports their application in studies of epithelial biology and reproductive physiology.

## 2 Materials and methods

### 2.1 Multimodal imaging strategy

To characterize epithelial organization across spatial scales, a multimodal imaging strategy was used. Brightfield and time-lapse imaging monitor organoid morphology and dynamic surface behavior. Confocal immunofluorescence microscopy was used to visualize cytoskeletal organization and epithelial architecture within intact organoids. Total internal reflection fluorescence (TIRF) microscopy provided selective excitation of fluorophores within approximately 100–200 nm of the glass interface, allowing visualization of actin structures localized to the surface in contact with the coverslip (Axelrod, 2001; Ellefsen et al., 2015; Zhang, 2021; de Medeiros et al., 2022). Scanning and transmission electron microscopy (SEM and TEM) were used to resolve ultrastructural features of epithelial surfaces and cell junctions at nanometer resolution (Hirokawa et al., 2009). Together, these complementary modalities were used to compare structural features associated with ECM-embedded apical-in and apical-out organoid states across imaging scales. Apical-in and apical-out structural states were interpreted based on the combined assessment of lumen orientation, epithelial morphology, and cortical F-actin distribution across imaging modalities. For imaging-based analyses, organoids were collected from four wells per condition and pooled prior to fixation and staining. The resulting organoid pellet was used for imaging across modalities. Because organoids were processed and imaged as pooled samples, the exact number of organoids visualized per image varied; however, multiple organoids were present in each preparation. Representative images were selected based on clarity and their ability to illustrate structural features consistently observed across samples. These imaging experiments were performed across two independent biological replicates. No formal statistical comparisons were performed because the imaging analyses were primarily descriptive and aimed at comparing structural features across modalities.

### 2.2 Organoid culture and polarity inversion

Bovine oviductal organoids were established using previously described protocols from our laboratory for organoid culture in ECM scaffolds (Thompson et al., 2022; Menjivar et al., 2026; Thompson-Brandhagen et al., 2026). Briefly, under standard ECM-embedded culture conditions, luminal cell clusters were embedded within 25 µL domes of UltiMatrix® (ECM; extracellular matrix; R&D Systems; Minneapolis, MN, United States), ECM was used at full manufacturer-supplied concentration without dilution, in 48-well culture plates (Corning, Corning, NY, United States; Cat. no. 3548) and maintained in complete organoid culture medium at 37 °C in a humidified incubator with 5% CO_2_, and half of the culture medium was replaced every 2–3 days. Complete organoid culture medium was prepared as previously described by Thompson-Brandhagen et al. (2026), including all supplements and growth factors required for bovine oviductal organoid maintenance.

**FIGURE 1:**
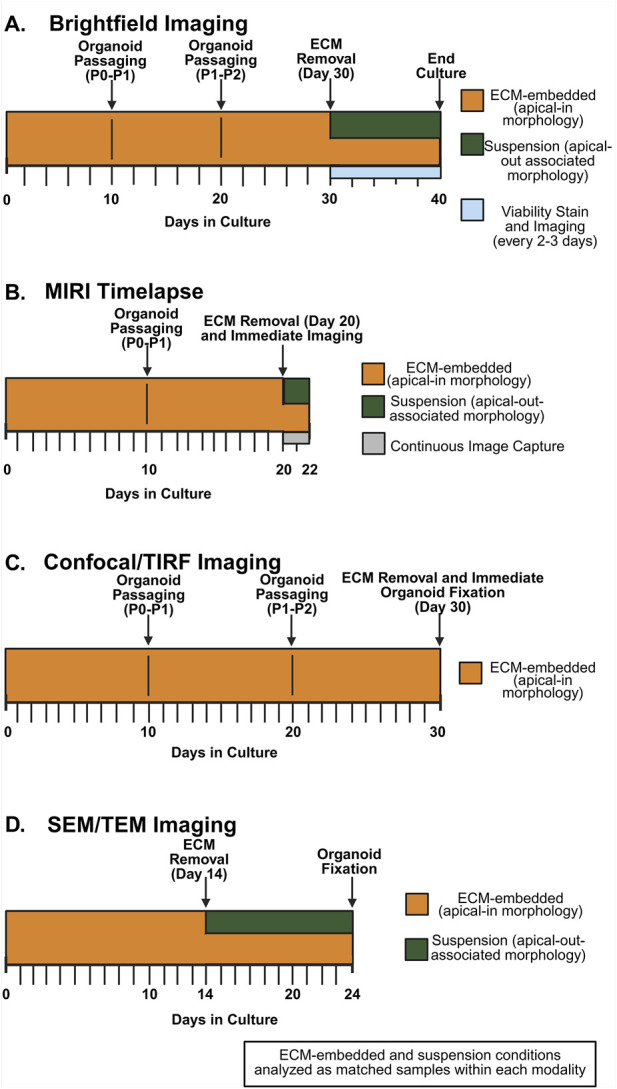
Experimental timeline for organoid culture, polarity reversal, and imaging across modalities. Schematic representation of bovine oviductal organoid culture timelines used for each imaging modality **(A)** Brightfield imaging workflow. Organoids were maintained in ECM culture (apical-in configuration) across passages (P0–P2), followed by ECM removal on Day 30 and transfer to suspension culture for an additional 10 days (40 days total) (apical-out conditions). Viability staining and morphological assessment were performed at defined time points following ECM removal **(B)** Time-lapse imaging workflow. Organoids were maintained in ECM culture for 20 days prior to ECM removal and transfer to suspension culture, followed by continuous image acquisition over 48 h (22 days total) **(C)** Confocal and TIRF imaging workflow. Organoids were maintained in ECM culture for 30 days with passaging and subsequently removed from the ECM for imaging. Apical-in and apical-out organoids were prepared as matched conditions and immediately fixed for imaging following sample preparation (<1 h post-ECM removal) to minimize secondary structural changes **(D)** SEM and TEM imaging workflow. Organoids were generated in ECM culture and maintained for 14 days. Organoids were then maintained in ECM culture or transferred to suspension culture following ECM removal and cultured for an additional 10 days prior to fixation and ultrastructural analysis (24 days total). Across all workflows, ECM culture (orange) represents apical-in conditions, whereas suspension culture (green) represents apical-out structural states. Imaging modalities were applied to matched samples within each condition to enable comparison of epithelial organization across spatial scales.

**FIGURE 2:**
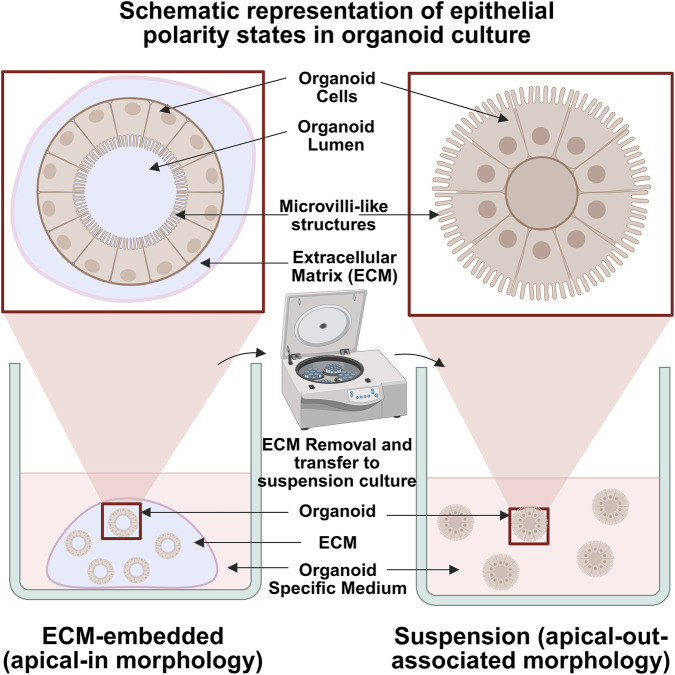
Schematic representation of epithelial organization in apical-in and apical-out organoid configurations. In ECM-embedded culture (left), organoids adopt an apical-in configuration in which the apical membrane faces a central lumen and the basolateral membrane interfaces with the surrounding extracellular matrix (ECM). Following ECM removal and transfer to suspension culture (center), organoids undergo structural reorganization, characterized by outward-facing epithelial surfaces. In suspension culture (right), organoids exhibit features consistent with an apical-out configuration, in which the actin-rich apical domain and associated surface specializations are oriented toward the external environment. This schematic illustrates canonical epithelial organization and polarity-associated structural states.

Organoids were maintained in ECM culture for passages 0–2 (P0-P2), with each passage cultured 10–14 days prior to experimental use (Figure 1). Under these conditions, organoids exhibited an apical-in configuration. Organoids with polarity reorganization were generated using protocols described previously by our laboratory (Thompson-Brandhagen et al., 2026). Briefly, organoids were removed from the ECM using Cell Recovery Solution (Corning Inc., Corning, NY, United States), a non-enzymatic reagent that depolymerizes basement membrane matrices, for 45 min at 4 °C, followed by two washes in 1 mL sterile phosphate-buffered saline (PBS) to remove residual matrix components. Organoids were then collected by centrifugation at 300 *g* for 1 min and resuspended in complete culture medium (Figure 2). Organoids were transferred to standard non-treated culture plates and maintained in suspension culture in 250 µL of standard organoid medium per well at 37 °C and 5% CO_2_, and with medium replaced every 2–3 days. Organoid density varied slightly between preparations due to pooling from multiple wells. To minimize aggregation, organoids were gently pipetted twice daily using wide-bore tips with approximately 2–3 pipetting cycles per handling session to minimize organoid aggregation while limiting mechanical disruption. Aggregation was monitored qualitatively during suspension culture but was not formally quantified. Polarity-associated structural features were assessed immediately following ECM removal and during subsequent suspension culture using brightfield and imaging-based analyses. For imaging experiments, apical-in and apical-out organoids were analyzed as matched conditions within each modality. Matching was based on organoids analyzed at comparable passage numbers, culture durations, and imaging timepoints. Where possible, organoids of similar overall morphology and size were selected for comparative imaging analyses.

For live- and fluorescence-based imaging modalities, including MIRI time-lapse imaging, confocal microscopy, and total internal reflection fluorescence (TIRF) microscopy, organoids were imaged immediately after sample preparation to capture polarity-defined structural states while minimizing post-processing effects. Apical-in organoids were imaged directly from ECM-embedded cultures, whereas apical-out organoids were imaged following ECM removal, centrifugation, and transfer to suspension culture as described above. This approach enabled comparison of polarity states without additional culture time that could introduce secondary structural changes. Additional imaging methods, such as scanning electron microscopy (SEM), transmission electron microscopy (TEM), and brightfield microscopy, were used to assess structural and ultrastructural features across polarity states. Because polarity-associated structural changes were assessed descriptively rather than as a kinetic endpoint, variability in inversion timing between individual organoids was not formally quantified.

### 2.3 Brightfield imaging

Brightfield microscopy was used to monitor organoid morphology under ECM-embedded (apical-in) and suspension (apical-out) conditions. Morphological assessments were performed at defined intervals during experiments, and representative images were collected immediately following sample preparation (apical out; Figure 1A). For time-lapse brightfield imaging, organoids were imaged using the MIRI time-lapse incubator (Esco Medical), with images acquired at 5-min intervals over a 48-h period (Figure 1B).

Organoid viability was assessed using trypan blue exclusion. Organoids were collected daily over a 10-day period, incubated briefly in 0.4% trypan blue solution, and immediately evaluated by brightfield microscopy. (Figure 1A). Organoids excluding dye were classified as viable, while those exhibiting dye uptake were classified as non-viable. Because trypan blue penetration in intact three-dimensional structures may be limited, this approach was used to approximate membrane integrity at the organoid level rather than to provide a quantitative assessment of viability within internal cell layers.

### 2.4 Time-lapse live imaging

For continuous live-cell imaging, organoids were imaged using the MIRI® Time-Lapse Incubator system (Esco Medical, Singapore). Two organoid configurations were prepared. For ECM-embedded (apical-in) organoids, bovine oviduct luminal cell clusters were seeded directly into 5 µL ECM domes in the center of wells of MIRI® Culture Coin dishes using the same procedure as standard ECM-embedded organoid culture (Menjivar et al., 2023; Thompson-Brandhagen et al., 2026), except that smaller ECM droplets were used to accommodate the imaging format. Organoids were maintained in standard organoid culture medium (Menjivar et al., 2023; Thompson-Brandhagen et al., 2026). For apical-out organoids, a subset of organoids from standard ECM-embedded apical-in cultures was removed from ECM using the matrix removal procedure described above (Section 2.2) and transferred to suspension culture in the wells of MIRI® Culture Coin dishes prior to imaging. Organoids were suspended in standard culture medium and maintained in the MIRI® Time-Lapse Incubator under controlled conditions (37 °C, 5% CO_2_, regulated humidity). Time-lapse imaging experiments were performed using 14 wells per plate across three plates per treatment condition. The number of organoids present within each well varied between preparations. Representative fields containing multiple organoids were monitored throughout imaging experiments. Time-lapse imaging was performed for 48 h using transmitted light microscopy at 5-min intervals, with images acquired via automated interval imaging controlled by MIRI software (Figure 1B).

### 2.5 Immunofluorescence and confocal microscopy

For immunofluorescence analysis, organoids in ECM culture were removed from the ECM using the matrix removal procedure described above (Section 2.2). The organoids were then washed twice in phosphate-buffered saline (PBS) (1 mL) and pelleted by centrifugation (3,000 × g for 10 min) to facilitate collection following matrix dissolution. Organoids were subsequently divided into two groups for fixation. One group was fixed immediately in 4% paraformaldehyde for 30 min at room temperature. The second group underwent a brief, low-speed centrifugation step (300 × g for 1 min) prior to fixation to promote settling and consistent orientation of organoids for imaging (Figure 1C).

Fixed organoids were permeabilized with 0.1% Triton X-100 in Tris-buffered saline (TBS) and incubated at 37 °C for 15 min, and then blocked in 5% goat serum in 0.1% Triton X-100 in TBS for 1 h. Organoids were incubated with Alexa Fluor™ 488 phalloidin (Thermo Fisher Scientific; Catalog #A12379) to label filamentous actin (F-actin), diluted in blocking buffer according to the manufacturer’s instructions, for 1 h at room temperature with gentle agitation. DAPI was used as a nuclear counterstain. Organoids were stained in approximately 4 µL staining solution per sample with gentle agitation throughout incubation steps. Samples were washed again before imaging. As with other three-dimensional organoid systems, incomplete penetration of fluorescent probes into deeper cellular layers cannot be fully excluded and represents a limitation of whole-organoid staining approaches.

Imaging was performed with an inverted laser-scanning confocal microscope (ZEISS LSM 980 Airyscan; Carl Zeiss Microscopy, Oberkochen, Germany) and a ×10 objective, with consistent acquisition settings across experimental groups in the Ghosh Laboratory (Colorado State University). Alexa Fluor 488 phalloidin and DAPI were imaged using standard 488 nm and 405 nm excitation laser lines, respectively. Z-stack image series were acquired to capture the full three-dimensional architecture of each organoid. For ECM-embedded (apical-in) organoids, z-stacks were acquired using a step size of 1.5 μm across 39 optical sections. For suspension-cultured (apical-out) organoids, z-stacks were acquired using a step size of 1.5 μm across 26 optical sections. The reduced stack depth in suspension-cultured organoids reflects differences in organoid morphology, including loss or collapse of the central lumen and a corresponding decrease in overall structure thickness following ECM removal. These parameters were selected to ensure adequate axial sampling while maintaining consistent acquisition settings across conditions. Orthogonal and maximum-intensity projections were generated to visualize the spatial localization of cortical actin relative to the organoid lumen or external surface (de Medeiros et al., 2022). Image processing and analysis were performed using ImageJ software (National Institutes of Health, Bethesda, MD, United States). Image analysis was performed qualitatively to assess overall structural organization and cortical F-actin distribution. Formal segmentation, threshold-based quantification, or morphometric polarity analysis was not performed.

### 2.6 Total internal reflection fluorescence (TIRF) microscopy

Organoids were prepared as described above (Section 2.5) and stained with Alexa Fluor™ 488 phalloidin. Stained organoids were transferred to glass-bottom imaging dishes (P35G-1.5-14-C, MatTek) and allowed to settle onto the coverslip surface before imaging. Because TIRF excitation is limited to fluorophores within approximately 100–200 nm of the glass interface, imaging was restricted to regions of the organoid in direct contact with the coverslip. To facilitate this interaction, organoids were allowed to settle naturally onto the coverslip surface prior to imaging and excess medium was gently removed to promote settling onto the imaging surface. As a result, TIRF microscopy was used to assess actin structures localized to the surface contacting the substrate, rather than to visualize the full three-dimensional architecture of the organoid.

TIRF imaging was performed on a custom-built ring TIRF microscope. Briefly, a 488 nm solid-state laser (OBIS 488, Coherent) was used for excitation. The ring TIRF configuration directs the excitation beam through a two-axis galvanometer mirror system (GVSK2-US0, Thorlabs) driven with sinusoidal and cosinusoidal waveforms, tracing a ring at the back focal plane of the objective and producing TIRF excitation from all 360° of incidence angles, thereby eliminating the uneven illumination inherent to single-angle TIRF (Fiolka et al., 2008; Ellefsen et al., 2015). Images were acquired using a 60× NA 1.50 oil-immersion objective (Olympus) and an EMCCD camera (iXon Ultra 888, Andor). A 300 mm achromatic doublet tube lens (AC254-300-A-ML, Thorlabs) was used in place of the standard 180 mm Olympus tube lens, yielding an effective magnification of ×100 and a pixel size of 130 nm. Fluorescence emission was collected through a 510/42 nm BrightLine bandpass filter (Semrock). Image acquisition was controlled using Micro-Manager (v2.0.1) with a custom Python interface based on Pycro-Manager (Pinkard et al., 2021), with hardware synchronization and galvanometer waveform generation handled by an Arduino Mega board.

### 2.7 Electron microscopy

#### 2.7.1 Sample fixation

A subset of organoids cultured in ECM was removed on day 14. At this time point, half of the organoids were maintained in ECM culture, while the remaining organoids were removed from ECM using Organoid Harvesting Solution (R&D Systems, Minneapolis, MN, United States) to depolymerize the matrix. Following matrix removal, organoids were washed in PBS by centrifugation (3,000 × g, 10 min) and gently centrifuged (300 × g for 1 min) to facilitate handling prior to transfer to suspension culture for 10 days (Figure 1D). During suspension culture, organoids were pipetted gently using wide-bore tips to prevent aggregation and maintain individual structures. After this period, organoids from both conditions, those maintained in ECM for 24 days and those cultured in suspension for 10 days following ECM removal, were collected and fixed in 2% glutaraldehyde prepared in 0.1 M cacodylate buffer (pH 7.4) containing 0.15 M sucrose for 2 h at room temperature. Following fixation, samples were stored in cacodylate buffer at 4 °C before processing for transmission or scanning electron microscopy. Samples were stored in cacodylate buffer at 4 °C prior to processing as described in the original publication (Thompson-Brandhagen et al., 2026).

#### 2.7.2 Scanning electron microscopy (SEM)

Samples fixed as described above were processed for SEM at the Electron Microscopy Core Facility at the University of Colorado Anschutz Medical Campus (Aurora, CO, United States), following standard preparation protocols for surface imaging. After primary fixation, samples were rinsed three times in 0.1 M cacodylate buffer and post-fixed for 1.5 h in 1% osmium tetroxide and 0.8% potassium ferrocyanide in 0.1 M cacodylate buffer. Samples were subsequently rinsed five times in distilled water and dehydrated through a graded ethanol series. Following dehydration, samples were transitioned to hexamethyldisilazane (HMDS). After a final incubation in 100% HMDS, excess HMDS was carefully removed, and samples were allowed to air-dry overnight at room temperature. Dried specimens were mounted onto stubs and imaged using either an Apreo SEM (Thermo Fisher Scientific) or a Sigma SEM (Zeiss). SEM analysis enabled high-resolution visualization of the external epithelial surface, including microvillar projections at the apical membrane. Images were acquired under low accelerating-voltage conditions (0.5 kV) with a beam current of approximately 25 pA and a working distance of ∼3.9–4.0 mm. A T1 detector was used for signal collection, optimized for high-resolution surface topography and cellular detail. Imaging parameters were selected to enhance surface contrast while minimizing charging and beam-induced damage. Representative images were selected from organoid preparations in which similar ultrastructural features were consistently observed across samples.

#### 2.7.3 Transmission electron microscopy (TEM)

Transmission electron micrographs included in this study were previously published and are reproduced with permission from *Cell and Tissue Research* (Thompson-Brandhagen et al., 2026). Detailed sample preparation and imaging protocols are described in the original publication. Briefly, bovine oviductal organoids cultured within ECM and those maintained in suspension for 10 days following ECM removal were fixed in 2% glutaraldehyde prepared in 0.1 M cacodylate buffer (pH 7.4) containing 0.15 M sucrose and stored in the same buffer at 4 °C. Semi-thin sections (∼1 μm) were prepared, mounted on glass slides, and stained with toluidine blue to assess organoid orientation prior to ultrastructural analysis. Samples were subsequently processed for TEM using standard procedures, including resin embedding, ultrathin sectioning, and electron microscopic imaging, as previously described. These methods enabled detailed characterization of epithelial ultrastructure, including apical specializations, junctional organization, and polarity-associated membrane orientation.

## 3 Results

### 3.1 ECM removal induces apical-out morphology while preserving organoid viability

**FIGURE 3:**
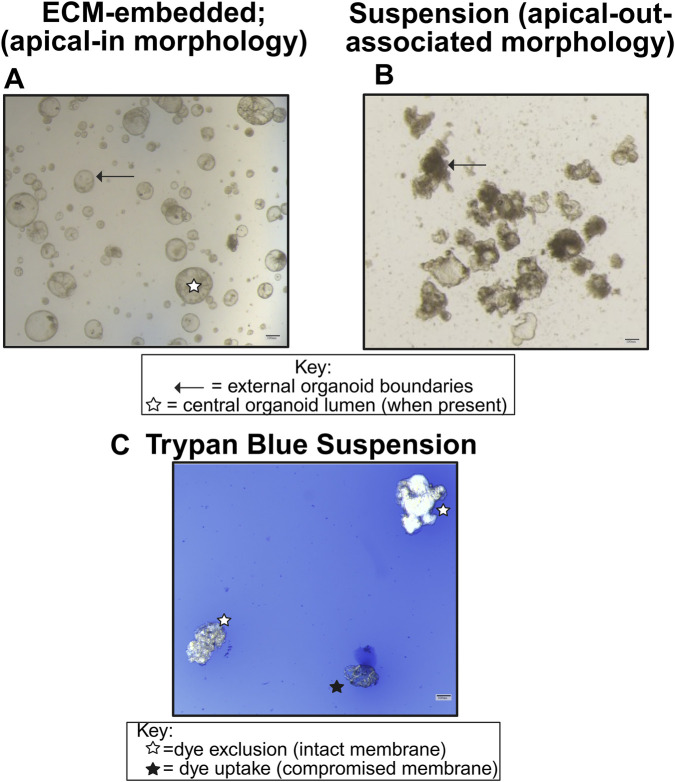
Brightfield morphology and viability assessment of bovine oviductal organoids under ECM-embedded and suspension conditions. **(A)** Representative brightfield image of organoids from a single well maintained in ECM culture, exhibiting spherical morphology with smooth external boundaries (arrow) and a clearly defined central lumen (star), consistent with apical-in structural organization. **(B)** Representative brightfield image of organoids from a single well following ECM removal and suspension culture. Organoids display compact, irregular morphology with scalloped outer boundaries (arrow) and frequently lack a clearly defined central lumen, consistent with previously described apical-out-associated structural features. Morphological criteria are used to describe structural states. **(C)** Trypan blue exclusion assay of organoids following ECM removal. Organoids excluding dye (white stars) retain membrane integrity, while organoids exhibiting dye uptake (black star) show compromised membrane integrity. Because dye penetration in intact three-dimensional structures may be limited, this assay provides an approximate assessment of organoid-level viability. Data shown are representative of n = 200 organoids across 2 biological replicates. Scale bars = 100 µm.

Under standard ECM-embedded culture conditions, bovine oviductal luminal cells consistently formed spherical organoids with a clearly defined central lumen (Figure 3A). Brightfield microscopy revealed smooth external contours and uniform epithelial organization surrounding the lumen. Following removal of ECM support, organoids exhibited altered morphology, adopting more compact and irregular structures that frequently lacked a clearly defined central lumen.

To assess the effects of ECM removal on organoid morphology and viability, organoids were transferred from ECM to suspension culture and assessed after 24 h. Following ECM removal and transfer to suspension culture, organoids displayed reproducible morphological changes, including loss of a clearly defined lumen and the emergence of compact epithelial structures with irregular, scalloped outer contours (Figure 3B). These features are consistent with previously described apical-out configurations, although morphology alone does not definitively establish apical-basal polarity (Figure 3).

At 24 h, 74% of organoids (n = 200, across 2 biological replicates) exhibited morphological features consistent with an apical-out configuration, while 26% retained features indicative of an apical-in morphology. Classification was performed descriptively based on brightfield morphological criteria, including lumen visibility and external contour characteristics, and was not conducted in a blinded manner. No intermediate timepoints were quantified. Assessment of organoid viability using trypan blue exclusion indicated high viability immediately following ECM removal and centrifugation (∼90%), with sustained viability at 72 h (∼85%) and a gradual decline during extended culture, reaching approximately 30% by day 10. These findings indicate that polarity-defined organoids remain structurally viable over a time window sufficient for imaging and short-term experimental applications (Figure 3C).

### 3.2 Live imaging reveals dynamic remodeling during polarity inversion

To assess the dynamic changes in organoid behavior, time-lapse imaging was performed under both ECM-embedded and suspension conditions over 48 h. Time-lapse imaging was used for a descriptive assessment of organoid structural behavior over time; formal quantitative tracking of polarity-inversion kinetics or morphometric changes was not performed. ECM-embedded cultures maintained a stable spherical morphology with smooth external contours and minimal movement (Supplementary Video S1). Following transfer to suspension culture, organoids exhibited increased movement within the medium, including rotational, lateral, and vertical displacement (Supplementary Video S2). In addition, structural changes were observed over time, including the development of irregular surface contours and transient shape changes, such as periodic expansion and contraction. These observations are descriptive and do not resolve the underlying mechanisms driving these changes.

### 3.3 Confocal microscopy reveals redistribution of cortical F-Actin

**FIGURE 4:**
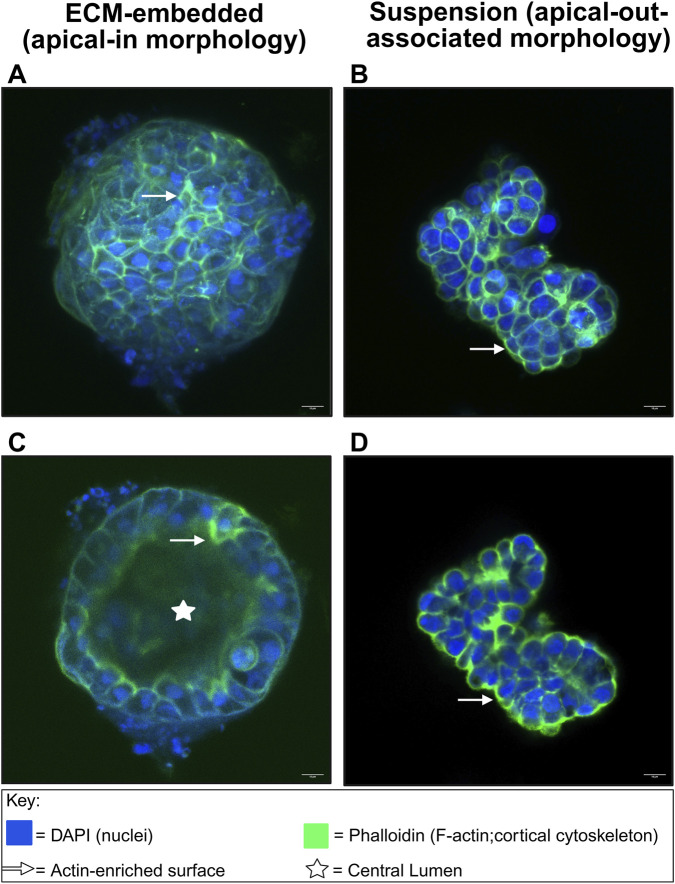
Confocal imaging of cytoskeletal organization in ECM-embedded and suspension-cultured organoids. **(A,C)** Representative confocal images of organoids maintained in ECM culture, exhibiting spherical morphology with a central lumen (star). F-actin (phalloidin, green) is enriched along the inward-facing luminal surface (arrows), while nuclei are labeled with DAPI (blue). **(B,D)** Representative confocal images of organoids following ECM removal and suspension culture. Organoids exhibit altered morphology with F-actin signal localized along the external surface of epithelial structures (arrows) and reduced or absent central lumen. F-actin is shown as a structural marker of cortical cytoskeletal organization. Redistribution of cortical F-actin is observed between conditions and is consistent with the reorganization of epithelial structure. Images represent maximum intensity projections and optical cross-sections from z-stack acquisitions. Scale bars = 10 µm.

To examine cytoskeletal organization within organoids, samples were analyzed by confocal microscopy following staining with phalloidin (F-actin) and DAPI. In ECM-embedded organoids, F-actin signal was enriched along the inward-facing luminal surface (Figure 4A). Optical cross-sections demonstrated a central lumen lined by actin-rich apical membranes, with nuclei organized in a continuous epithelial layer (Figure 4C). In organoids cultured following ECM removal, F-actin localization differed, with signal detected along the external surface of epithelial structures rather than within a clearly defined lumen (Figure 4B). These organoids frequently lacked a well-defined central lumen and instead exhibited actin-rich cortical structures outlining the external boundary of the epithelial aggregates (Figure 4D).

These findings indicate a redistribution of cortical F-actin, consistent with the reorganization of the epithelial structure. However, because F-actin localization alone does not uniquely define apical-basal polarity, these observations are interpreted as structural features associated with polarity-defined states rather than definitive evidence of polarity orientation.

### 3.4 TIRF microscopy findings are consistent with surface accessibility of the apical surface

**FIGURE 5:**
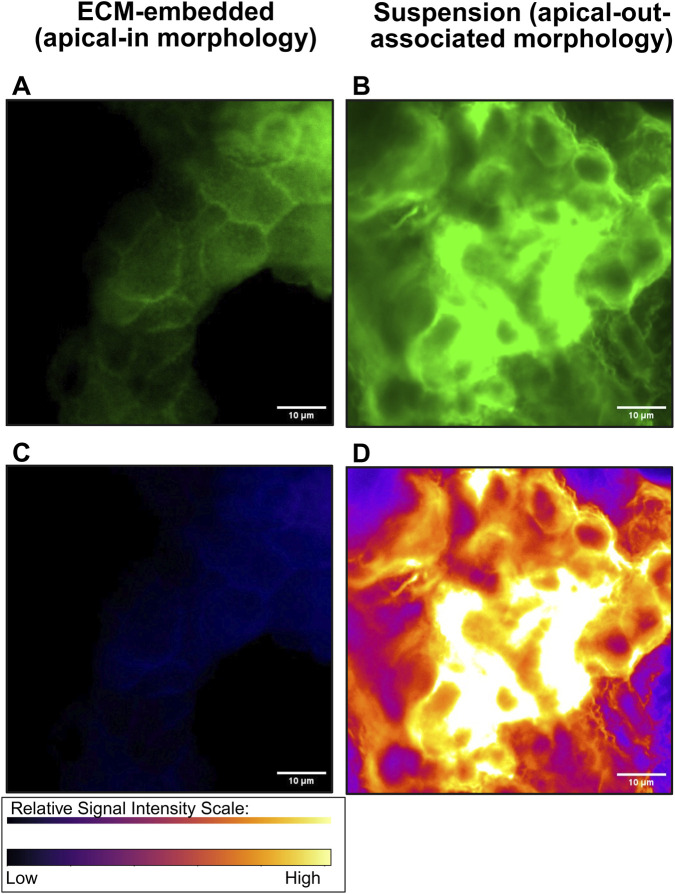
TIRF microscopy of surface-associated F-actin in organoids under ECM-embedded and suspension conditions **(A,C)** Representative TIRF images of organoids maintained in ECM culture. Due to the limited penetration depth of TIRF excitation (∼100–200 nm), the signal is restricted to fluorophores located within the region of the organoid in direct contact with the coverslip. Under these conditions, the phalloidin signal is minimal within the evanescent field, consistent with the limited accessibility of actin-rich structures at the imaging interface. **(B,D)** Representative TIRF images of organoids following ECM removal and suspension culture. An increased phalloidin signal is detected within the evanescent field, indicating the presence of actin structures at the surface interacting with the coverslip. **(C,D)** Corresponding signal-intensity renderings illustrate relative differences in surface-proximal F-actin signal across conditions. Because TIRF imaging selectively visualizes only the region of the sample in contact with the coverslip, signal intensity reflects surface accessibility and positioning rather than global three-dimensional organization. As such, these observations support the presence of differences in surface-associated actin structures. Images were acquired using identical acquisition and processing settings. Scale bars = 10 µm.

To further assess surface-associated actin structures, phalloidin-labeled organoids were imaged using TIRF microscopy. Because TIRF excitation is restricted to fluorophores within approximately 100–200 nm of the coverslip interface, signal detection is limited to structures in direct contact with the imaging surface. In ECM-embedded organoids, phalloidin signal within the evanescent field was minimal, consistent with limited accessibility of actin-rich surfaces at the coverslip interface (Figure 5A). In contrast, organoids cultured following ECM removal exhibited increased phalloidin fluorescence signal within the evanescent field, indicating the presence of actin structures at the surface interacting with the coverslip (Figure 5B). Heat-map intensity rendering further illustrates differences in surface-proximal F-actin signal between conditions (Figures 5C,D). These observations support differences in surface accessibility of actin-rich structures but do not independently establish apical-basal polarity.

### 3.5 Scanning electron microscopy reveals surface ultrastructural differences

**FIGURE 6:**
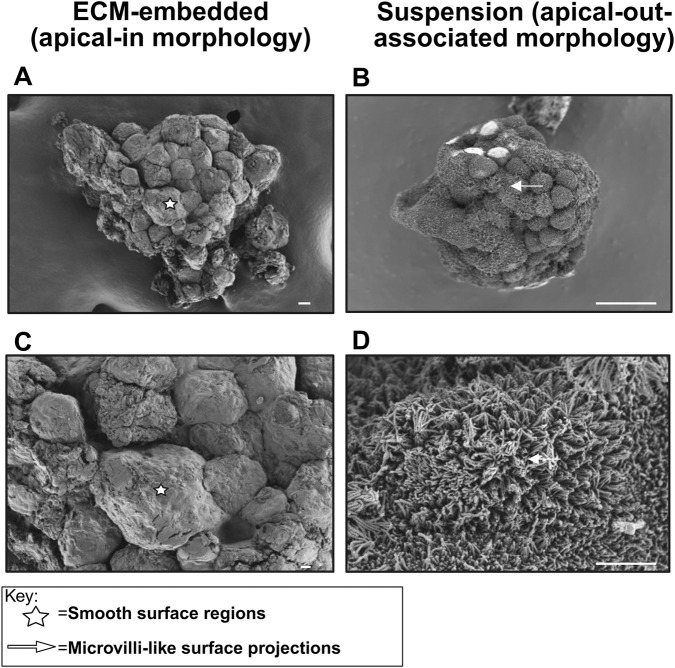
Scanning electron microscopy (SEM) of organoid surface topology under ECM-embedded and suspension conditions. **(A,B)** Low-magnification SEM images showing overall surface morphology of apical-in **(A)** and apical-out **(B)** organoids. SEM provides high-resolution visualization of surface topology by detecting secondary electrons emitted from the specimen surface, enabling detailed assessment of membrane architecture. Apical-in organoids exhibit relatively smooth external surfaces [**(A)** ★★]. In contrast, apical-out organoids display irregular, textured surfaces [**(B)** arrow]. **(C,D)** Higher-magnification SEM images highlighting surface ultrastructure. Apical-in organoids [**(C)** ★★] lack prominent surface projections. In contrast, apical-out organoids [**(D)** arrow] exhibit dense arrays of microvilli-like projections, characteristic of apical epithelial specializations. Scale bars: 20 µm **(A,B)** 3 µm **(C,D)**. Magnification varies across panels due to differences in the field of view and is therefore represented by scale bars rather than single values.

To evaluate surface ultrastructure at high resolution, organoids were examined using scanning electron microscopy (SEM). ECM-embedded organoids exhibited smooth external surfaces at low magnification (Figures 6A,C). In contrast, organoids cultured following ECM removal displayed irregular, textured external surfaces (Figure 6B). At higher magnification, dense hair-like projections were observed across the external surface of these organoids (Figure 6D), consistent with microvillar-like structures. These projections were not observed on the ECM-embedded organoids.

### 3.6 Transmission electron microscopy highlights polarity-associated features at the ultrastructural level

**FIGURE 7:**
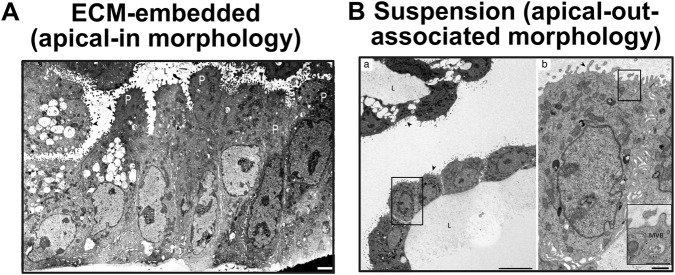
Transmission electron microscopy (TEM) of epithelial ultrastructure in organoids under ECM-embedded and suspension conditions. TEM enables nanoscale visualization of intracellular and membrane ultrastructure, providing evidence of epithelial polarity orientation through apical specializations, such as microvilli, relative to the lumen or the external environment. **(A)** TEM micrograph of bovine oviductal organoids cultured within extracellular matrix (apical-in orientation), demonstrating a contiguous pseudostratified epithelium with apical specializations directed toward a central lumen (L). Cells exhibit characteristic epithelial ultrastructure, including euchromatic nuclei, mitochondria, and secretory vesicles. Prominent microvilli are observed along the luminal (apical) surface. Peg cells (P), identified by increased electron density, are interspersed within the epithelium and contribute to the pseudostratified organization. Scale bars: 2 μm. **(B)** TEM micrographs of organoids cultured in suspension (apical-out orientation). In contrast to apical-in organoids, microvilli (arrowheads) are localized exclusively to the external surface, supporting outward orientation of apical specializations (a, left). Luminal spaces (L) may still be present in some regions; these are less prominent and lack the continuous organization observed in ECM-embedded organoids. Higher-magnification views (b, right) reveal preserved cellular ultrastructure, including intact mitochondria and multivesicular bodies (MVB; inset), indicative of maintained cellular viability and function following polarity reversal. Scale bars: A = 10μm; B = 1μm; inset = 600nm. Images reproduced and adapted with permission from Thompson-Brandhagen et al. [Journal of Cell and Tissue Research/Thompson-Brandhagen et al., 2026].

Organoids were analyzed by transmission electron microscopy (TEM) to examine epithelial organization at a higher resolution. In ECM-embedded organoids, epithelial cells formed a continuous layer surrounding a central lumen with microvilli projecting into the luminal space and junctional complexes localized near the apical region (Figure 7A). In organoids cultured following ECM removal, epithelial organization differed in orientation, with apical specializations observed at the external surface (Figure 7B). Although internal luminal spaces were still present in some organoids, these were often less prominent and lacked the organized, lumen-facing architecture observed in ECM-embedded organoids. Instead, apical features were observed at the periphery of the epithelial aggregates, consistent with reorganization of epithelial architecture (Figure 7B).

## 4 Discussion

Access to the oviductal epithelium *in vivo* is limited by anatomical constraints, yet this tissue plays a central role in fertilization, early embryonic development, and embryo transport (Coy et al., 2012; Maillo et al., 2016; Rizos et al., 2016). Organoid systems provide a powerful *in vitro* platform to model this environment; however, conventional ECM-embedded organoids adopt an apical-in configuration that restricts direct access to the luminal epithelial surface (Rossi et al., 2018; Clevers, 2016). This presents a practical limitation for studying processes at the epithelial interface, including secretion, extracellular vesicle signaling, and interactions with gametes and embryos (Lopera-Vasquez et al., 2017; Almiñana and Bauersachs, 2019).

In this study, removal of ECM followed by centrifugation was associated with reproducible structural reorganization of bovine oviductal organoids, resulting in features consistent with an apical-out configuration while maintaining overall structure and short-term viability. These observations are consistent with prior reports demonstrating polarity plasticity in epithelial organoid systems following disruption of matrix-derived cues (Akhtar and Streuli, 2013; Co et al., 2019). Here, ECM-embedded organoids (apical-in) and organoids cultured following ECM removal (apical-out) were used as defined structural models to compare how different imaging methods visualize epithelial organization. Rather than focusing on the mechanisms or kinetics of polarity reorganization, this study uses polarity-defined states as a framework to evaluate modality-dependent interpretations of epithelial structure.

Across imaging methods, consistent structural differences were observed between ECM-embedded organoids and organoids cultured after ECM removal (Figures 3–7). Within each modality, these comparisons demonstrate that interpretation of epithelial organization is influenced by polarity-defined structural state. Importantly, these observations are based on structural features rather than direct molecular confirmation of polarity orientation and should therefore be interpreted as reflecting polarity-associated organization rather than definitive assignment of apical-basal identity. A key finding is that no single imaging modality fully resolves epithelial organization in three-dimensional systems. Brightfield imaging enabled rapid assessment of global morphology but lacked the resolution needed to determine membrane orientation. Live imaging revealed differences in organoid behavior and surface dynamics, but the findings remained primarily descriptive. Quantitative morphodynamic analyses, including tracking of displacement, rotational behavior, or shape metrics, were beyond the scope of the present descriptive imaging study. Confocal microscopy provided spatial resolution of cytoskeletal organization within intact structures, while TIRF microscopy enabled visualization of actin structures at the imaging surface. Electron microscopy further extended these observations by resolving ultrastructural features, including microvillar projections and junctional organization. Together, these findings highlight that each modality provides distinct and complementary information, and that interpretation of epithelial organization benefits from integration across imaging scales (Bryant and Mostov, 2008; Rodriguez-Boulan and Macara, 2014).

Filamentous actin is enriched at the apical domain of epithelial cells and is widely used in organoid systems as a structural readout of membrane organization. Consistent with prior studies, redistribution of cortical F-actin was observed following ECM removal (Fatehullah et al., 2013; Rijns et al., 2025; Tidball et al., 2025; Medvedeva et al., 2026). However, because F-actin localization alone does not uniquely define apical-basal polarity, these findings are interpreted in conjunction with morphological and ultrastructural features rather than as standalone evidence of polarity orientation.

Polarity-associated structural changes were observed across multiple imaging modalities following ECM removal, demonstrating that these features can be captured within a short time window without prolonged culture. Although some structural changes may also reflect adaptive responses to ECM removal and suspension culture, the consistent organization observed across imaging modalities supports interpretation of these features as polarity-associated structural states. Viability analysis further supports the practical utility of this system, indicating that organoids remain structurally intact immediately after processing and maintain viability for several days in suspension culture, providing a useful window for imaging and short-term experimental applications. However, interpreting these structural states should account for potential confounding factors associated with ECM removal and suspension culture, including mechanical stress and altered cell-matrix interactions, which may contribute to the observed morphological changes. These observations are consistent with previous work showing that epithelial organization in organoid systems is responsive to microenvironmental cues, particularly interactions with the ECM (Akhtar and Streuli, 2013; Co et al., 2019; Kakni et al., 2022). In ECM-embedded cultures, integrin-mediated signaling and basement membrane interactions reinforce basolateral identity and stabilize inward-facing organization (Akhtar and Streuli, 2013; Rodriguez-Boulan and Macara, 2014). Disruption of these cues is associated with reorganization of epithelial architecture, reflecting the inherent plasticity of epithelial systems observed across multiple organoid models, including intestinal, biliary, and renal models (Parigoris et al., 2022; Fiorotto et al., 2023; Kakni et al., 2023; Yousef Yengej et al., 2023). This study extends these observations to bovine oviductal organoids by providing multiscale structural comparison in a reproductive context, where such analyses have been comparatively limited.

The ability to modulate epithelial orientation has important implications for studying oviductal biology and advancing assisted reproductive technologies. *In vivo*, the apical surface of the oviductal epithelium is the site of key physiological processes, including ciliary-driven fluid movement, secretion of regulatory factors, and direct interaction with gametes and early embryos (Coy et al., 2012; Maillo et al., 2016; Rizos et al., 2016; Menjivar et al., 2023). By increasing accessibility to this interface, apical-out organoids provide a system for investigating these processes under controlled conditions. This is particularly relevant for embryo co-culture applications, where interactions between the embryo and the oviductal epithelium influence developmental competence (Almiñana, 2015; Almiñana et al., 2017; Bondarenko and Turco, 2025). In addition, access to the apical surface enables investigation of epithelial-derived extracellular vesicles and luminal secretions that mediate embryo–maternal communication (Avilés et al., 2015; Almiñana et al., 2017; Lopera-Vasquez et al., 2017).

Although this study focuses on structural characterization, the preservation of epithelial organization and apical specializations suggests that structural features relevant to functional competence are maintained, although functional activity was not directly assessed (Hirokawa et al., 2009; Odenwald et al., 2017; Gjorevski et al., 2022). However, functional properties such as directional secretion, ciliary activity, and barrier integrity were not directly assessed. Future studies incorporating embryo co-culture, extracellular vesicle analysis, and dynamic culture systems will be important to establish the extent to which these structural states correspond to physiological function (Maillo et al., 2016; Pranomphon et al., 2024).

Beyond reproductive biology, these findings reinforce the concept that epithelial organization in organoid systems can be experimentally modulated by altering the extracellular environment. This expands the versatility of organoid platforms for studying epithelial biology, host–pathogen interactions, and tissue-specific physiology (Rossi et al., 2018; Kim et al., 2020; Bondarenko and Turco, 2025). Importantly, polarity-manipulated organoids differ from spheroid systems, which may also present outward-facing surfaces but typically lack the organized epithelial architecture, defined lineage composition, and tissue-specific differentiation characteristic of organoids. As a result, polarity-manipulated organoids retain key structural features of native epithelium while enabling experimental access to the luminal surface.

In the context of the oviduct, the ability to generate a viable, apical-out model provides a platform for investigating epithelial-luminal interactions that are otherwise difficult to access *in vivo*. Collectively, this study establishes a multiscale imaging framework for interpreting epithelial organization in organoid systems and identifies polarity-defined structural states as a potentially informative parameter for improving cross-modal interpretation. These findings support the use of bovine oviductal organoids as an accessible model for investigating epithelial organization and luminal interactions in reproductive biology.

## Data Availability

The original contributions presented in the study are included in the article/Supplementary Material, further inquiries can be directed to the corresponding author.

## Supplementary material

Supplementary Video S1 Time-lapse imaging of apical-in bovine oviductal organoids. Brightfield time-lapse imaging of bovine oviductal organoids maintained in extracellular matrix (ECM) culture. Organoids retain a stable spherical morphology with smooth external contours over the imaging period. Minimal movement or structural change is observed, consistent with an apical-in configuration. Images were acquired at 5-min intervals over 48 h.

Supplementary Video S2 Time-lapse imaging of apical-out bovine oviductal organoids. Brightfield time-lapse imaging of bovine oviductal organoids following ECM removal and centrifugation, maintained in suspension culture. Organoids exhibit dynamic movement, including rotational, lateral, and vertical displacement, along with changes in surface morphology over time. These features are consistent with an apical-out configuration. Images were acquired at 5-min intervals over 48 h.
